# Using deep learning method to identify left ventricular hypertrophy on echocardiography

**DOI:** 10.1007/s10554-021-02461-3

**Published:** 2021-11-10

**Authors:** Xiang Yu, Xinxia Yao, Bifeng Wu, Hong Zhou, Shudong Xia, Wenwen Su, Yuanyuan Wu, Xiaoye Zheng

**Affiliations:** 1 Department of Cardiology, The Fourth Affiliated Hospital, School of Medicine, Zhejiang University, N1 Shangcheng Avenue, Yiwu, 322000 China; 2 Key Laboratory for Biomedical Engineering of Ministry of Education, Zhejiang University, Zheda Avenue, Hangzhou, 310027 China; 3 Department of Cardiology, The First Affiliated Hospital, School of Medicine, Zhejiang University, Hangzhou, 310006 China

**Keywords:** Left ventricular hypertrophy, Deep learning, Echocardiography

## Abstract

**Background:**

Left ventricular hypertrophy (LVH) is an independent prognostic factor for cardiovascular events and it can be detected by echocardiography in the early stage. In this study, we aim to develop a semi-automatic diagnostic network based on deep learning algorithms to detect LVH.

**Methods:**

We retrospectively collected 1610 transthoracic echocardiograms, included 724 patients [189 hypertensive heart disease (HHD), 218 hypertrophic cardiomyopathy (HCM), and 58 cardiac amyloidosis (CA), along with 259 controls]. The diagnosis of LVH was defined by two experienced clinicians. For the deep learning architecture, we introduced ResNet and U-net++ to complete classification and segmentation tasks respectively. The models were trained and validated independently. Then, we connected the best-performing models to form the final framework and tested its capabilities.

**Results:**

In terms of individual networks, the view classification model produced AUC = 1.0. The AUC of the LVH detection model was 0.98 (95% CI 0.94–0.99), with corresponding sensitivity and specificity of 94.0% (95% CI 85.3–98.7%) and 91.6% (95% CI 84.6–96.1%) respectively. For etiology identification, the independent model yielded good results with AUC = 0.90 (95% CI 0.82–0.95) for HCM, AUC = 0.94 (95% CI 0.88–0.98) for CA, and AUC = 0.88 (95% CI 0.80–0.93) for HHD. Finally, our final integrated framework automatically classified four conditions (Normal, HCM, CA, and HHD), which achieved an average of AUC 0.91, with an average sensitivity and specificity of 83.7% and 90.0%.

**Conclusion:**

Deep learning architecture has the ability to detect LVH and even distinguish the latent etiology of LVH.

**Supplementary Information:**

The online version contains supplementary material available at 10.1007/s10554-021-02461-3.

## Introduction

Left ventricular hypertrophy (LVH) is a common cardiac morphology change caused by many different diseases such as hypertension, hypertrophic cardiomyopathy (HCM), cardiac amyloidosis (CA), etc. And it is an independent risk factor for other cardiovascular events [1]. If left untreated, as the disease progresses and worsens, it will lead to cardiac dysfunction, arrhythmia, and eventually heart failure or sudden death [2–4]. Therefore, it is important to detect LVH and accurately identify the cause in the early stages of the disease. Echocardiography, due to its easy accessibility and availability, is an essential non-invasive and non-radioactive diagnostic modality used to assess the changes of left ventricular (LV) structure [5] and is also a reliable method to identify LVH [6]. However, the result is highly dependent on the operator’s experience and may vary between echocardiographers, especially for new and inexperienced sonographers. Inter- and intra-operator variability is a common problem in echocardiography [7, 8]. Therefore, there is a need to develop an objective and automatic diagnostic system to assist clinicians in identifying LVH. Deep learning, an advanced machine learning method, can automatically extract features from large data sets to significantly improve the performance of tasks such as visual object recognition and object detection [9]. In recent years, it has been extensively applied to medical image recognition research and proved to be a promising approach to tackle different medical image process tasks [10, 11]. Numerous studies have investigated the performance of deep learning in the echocardiography field [12, 13], such as view classification, cardiac function evaluation, disease detection, etc., and have shown exciting results [14, 15]. Therefore, in this study, we believe that it is viable to utilize deep learning algorithms to detect LVH and identify the etiology in three types of diseases, HCM, CA, and hypertensive heart diseases (HHD).

## Methods

### Study population

Data collection for this study was approved by the Institutional Review Board of The First Affiliated Hospital, School of Medicine, Zhejiang University. Patients with LVH were identified by searching the echocardiography database of The First Affiliated Hospital, School of Medicine, Zhejiang University for reports including the keyword “left ventricular hypertrophy” between January 2018 and December 2020. An experienced echocardiographer and a cardiologist (both with over ten years clinical experience) defined the diagnosis of LVH and etiology through examination reports and corresponding clinical data. LVH was defined as LV mass index (LVMI) > 95 g/m^2^ in women and LVMI > 115 g/m^2^ in men by linear method, except for apical HCM,which may have normal LVMI. The diagnostic criteria of the three diseases were: (1) HHD: patients were diagnosed with a combination of hypertension and LV wall thickness confirmed by echocardiography, with no other conditions of LVH [16]. (2) HCM: patient’s maximal end-diastolic LV wall thickness ≥ 15 mm and no other causes of LVH [17]. (3) CA: suspicion of CA through echocardiography, confirmed amyloidosis by tissue biopsy, or confirmed by late gadolinium enhancement cardiac magnetic resonance (CMR) imaging [18]. We also included patients with normal cardiac structure (without LVH) as the control group.

### Echocardiography

Two standard views of end-diastolic, parasternal long-axis (PLX), and apical four-chamber (A4C) views of three types of LVH, including HHD, HCM, and CA, were collected, and another set of non-LVH images was obtained as the control group. These two-dimensional (2D) transthoracic echocardiograms were performed by experienced sonographer (3–10 years experiences) using two ultrasound machines (Vivid E9, GE Healthcare and EPIQ 7C, Philips). The images were stored in Digital Imaging and Communications in Medicine (DICOM) format with a resolution of 636*434 pixels (Vivid E9) or 800*600 pixels (EPIQ 7C). Due to disease progression or condition improvements, like changes in wall thickness or the emergence or disappearance of pericardial effusion, as well as the angle and position of the probe, echocardiograms of the same patient may vary at different inspection times. Therefore, images from the same patients at different times (more than one month apart) were also included. The treatment during these examination intervals did not significantly change wall thickness (from LVH to non-LVH).

### Datasets

We divided images into training, validation and testing sets (60:20:20). The images of the same patient were not distributed in different data sets considering that some patients who have undergone multiple examinations generated several pairs of images and the difference between these images was relatively small for humans. The annotations for the two cardiac views were created by author Yu. The labels for the three types of LVH were created by the consensus of author Wu and author Xia based on diagnostic criteria. The manually delineated LV myocardium were used as the ground truth for training the segmentation network. This process was performed on open annotation software Labelme. All Images were cropped to 384*384 pixels and converted to grey scale pictures. The images used to train the classification model were further normalized and resized to 224*224 pixels. We also applied image augmentation to the echocardiograms used in training the segmentation network, including random shifts of contrast, brightness, or saturation, with or without horizontal flips.

### Deep learning networks

**Fig.1:**
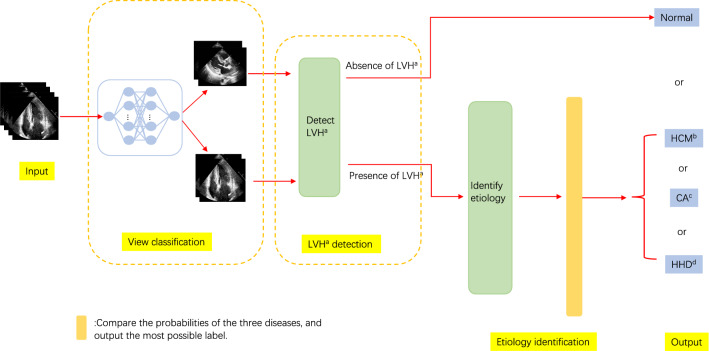
The architecture of the integrated framework. ^a^Left ventricular hypertrophy, ^b^hypertrophic cardiomyopathy, ^c^cardiac amyloidosis ^d^hypertensive heart disease

**Fig. 2:**
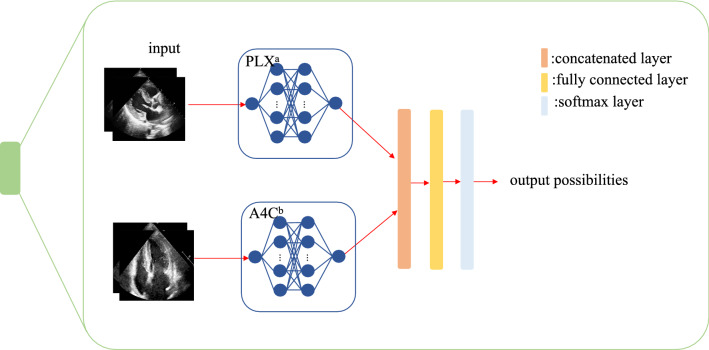
Basic architecture of LVH detection and etiology classification models. This figure shows the inner architecture of the green block in the Fig. 1. ^a^parasternal long-axis, ^b^apical four-chamber

We built a framework based on ResNet and Unet+ + that shows excellent performance in image recognition and segmentation [19, 20]. (1) Classification: We introduced ResNet to extract features and help solve three classification tasks: view classification, LVH detection, and etiology identification. View classification and LVH detection models were binary classification networks for separating two views (A4C and PLX) and discriminating the normal structure from LVH, respectively (Fig. 1). Then we constructed a three-class classification network to identify three types of LVH (HHD, HCM and CA) (Fig. 1). For the LVH and etiology classification models in our study, each model consists of two sub-networks that extracted features from A4C and PLX, respectively. We used a concatenated layer to merge the features of the two views and those would go through a fully connected layer and a softmax layer sequentially, then output the probabilities (rang from 0 to 1) of each class (Fig. 2). Considering the complexity, training time and computational consumption of the different tasks, we chose ResNet18 for view classification and ResNet50, which have deeper layers, for LVH detection and etiology identification. (2) Segmentation: To find out whether the framework can perform well with only LV myocardium, which contains the most important information about recognizing LVH, and whether the performance of the network would be improved with segmented images, we introduced and trained U-net++ to segment the LV myocardium using the manually delineated contours as the ground truth. The output masks will be combined with the original images to form new images which will be used to train the same classification networks.

We introduced cross-entropy loss as the loss function and Adam as optimizer of the networks. ReduceLROnPlateau, StepLR or Cosine Annealing LR were used to adjust the learning rate. Early stopping and L2 regularization were applied to avoid overfitting. We trained different networks separately and used the validation set to fine-tune the hyperparameters. After that, we selected the model with the best performance (mainly determined by accuracy as well as AUC) to construct our final framework (Fig. 1). The input image pairs (sets of A4C and PLX) are sequentially passed through the different parts of the integrated framework. Finally, the most possible label was output, i.e. Normal, HCM, HHD, or CA. For example, if one pair of images (A4C and PLX) of a patient was classified as “abnormal”, then the images will enter into the next model to identify the cause of LVH and output the most possible disease (such as HCM), otherwise, the output result is “Normal” (Fig. 1).

In order to make the networks more interpretable, we used Gradient-weighted Class Activation Mapping (Grad-CAM) approach to create heat maps that highlighted the region of interest in the echocardiography for predicting the labels. All networks were developed in Python 3.7, Pytorch 1.7.0, and Cuda 10.2. All models were trained with graphics processing units (GPUs; TITAN RTX, Nvidia).

### Comparison with echocardiographers

Two echocardiographers, with 4–5 years of clinical experience, analyzed the same images on the test dataset to identify LVH firstly, then to discriminate the possible etiology of LVH, same procedure as the deep learning network. The final results were determined by consensus.

### Statistical analysis

Dichotomous data are presented as percentages. Continuous variables are presented as mean ± standard deviation. Continuous variables between multiple groups were analyzed by ANOVA. Categorical variables were analyzed using the χ^2^ test. The performance of the classification networks was evaluated using accuracy, the receiver operating characteristic (ROC) curve, the area under the ROC curve (AUC), and the corresponding specificity and sensitivity calculated at the Youden Index, with a 95% confidence interval (CI). The Dice (Dice Similarity Coefficient), IOU (intersection over union) and 95%HD (95% Hausdorff distance) were used to evaluate the performance of the segmentation. Statistical analysis was performed using SPSS (version 26.0) and MedCalc (version 19.6.4). P < 0.05 was considered statistical significance.

## Results

The study population consisted of 724 patients who underwent 805 examinations, resulting in a total of 1610 echocardiography. Of these patients, 259 had normal wall thickness and the remaining patients were diagnosed with HHD (n = 189), HCM (n = 218) (including 37 apical hypertrophy), and CA (n = 58), respectively. Among these patients, 573 undertook examinations using Vivid E9 machine, another 151 using EPIQ 7C machine. A part of patients undertook several examinations, and the time interval between the examinations was 3.9 ± 3.0 months (ranging from 1 to 15 months).The details of the study population and their baseline characteristics are shown in Table 1. We split the dataset according to a 60:20:20 ratio, generating 964 images (432 patients) for the training set, 332 images (150 patients) for the validation set, and 314 images (142patients) for the test set. The baseline characteristics of training, validation and testing groups for each of the patient categories were shown in online Table S1-S4.The loss and accuracy curves of training and validation were in online Figs. 1, 2 and 3.

**Table 1:** Baseline characteristics of patients and measurements of echocardiography

	Normal	Hypertrophy cardiomyopathy	Hypertensive heart disease	Cardiac amyloidosis	P value
No. of Patient	259	218	189	58	–
No. of cases	259	220	189	137	–
Male	71.4%	66.1%	75.5%	77.5%	0.12
Age (years)	56.1 ± 15.4	56.7 ± 14.0	60.6 ± 12.9	61.0 ± 8.4	< 0.05
BSA (m^2^)	1.69 ± 0.14	1.69 ± 0.13	1.69 ± 0.15	1.68 ± 0.14	0.59
IVSd (cm)	0.96 ± 0.11	1.98 ± 0.54	1.32 ± 0.14	1.52 ± 0.22	< 0.05
LVPWd (cm)	0.93 ± 0.12	1.10 ± 0.22	1.15 ± 0.16	1.38 ± 0.27	< 0.05
IVSd/LVPWd	1.04 ± 0.12	1.86 ± 0.62	1.15 ± 0.10	1.11 ± 0.13	< 0.05
LVM (g)	148.7 ± 31.5	283.2 ± 86.0	223.8 ± 85.7	238.3 ± 61.8	< 0.05
LVMI	87.7 ± 16.9	167.1 ± 49.9	135.6 ± 44.6	142.1 ± 34.3	< 0.05
EF (%)	65.0 ± 6.7	69.5 ± 7.6	66.3 ± 8.8	58.1 ± 10.7	< 0.05
LVDd (cm)	4.61 ± 0.39	4.41 ± 0.52	4.74 ± 0.59	4.17 ± 0.42	< 0.05
LVOT (mmHg)	–	31.9 ± 43.22	–	–	
GLS-Avg (%)	–	–	–	12.93 ± 4.13	

*BSA* body surface area, *IVSd* diastolic interventricular septum, *LVPWd* diastolic left ventricular post wall, *LVM* left ventricular mass, *LVMI* left ventricular mass index, *EF* ejection fraction, *LVDd* diastolic left ventricular diameter, *LVOT* left ventricular outflow tract, *GLS-Avg* Average global longitudinal strain

**Fig. 3:**
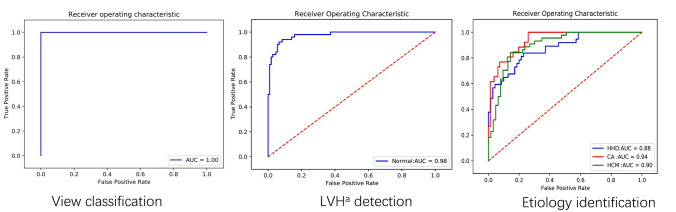
The results of individual classification network. *LVH* left ventricular hypertrophy, *HCM* hypertrophic cardiomyopathy, *CA* cardiac amyloidosis, *HHD* hypertensive heart disease

The results of the individual networks are shown below. First, we trained the network to differentiate two views (A4C and PLX). The AUC of this network was 1.0 (Fig. 3). Then, we developed a second network to distinguish LVH from non-LVH. This network performed well with AUC = 0.98 (95% CI 0.94–0.99) (Fig. 3), and corresponding sensitivity and specificity of 94.0% and 91.6%, respectively (Table 2). The accuracy of detecting LVH was 92.4%. Furthermore, to detect the latent causes of LVH, we trained a three-class classification network, and the AUC of the three diseases produced by the model on the testing set were 0.90 (95% CI 0.82–0.95) for HCM, 0.94 (95% CI 0.88–0.98) for CA, and 0.88 (95% CI 0.80–0.93) for HHD (Fig. 3) and the accuracy of this network was 75.7%. The specificity and sensitivity corresponding to the three diseases were shown in Table 2.

**Table 2:** Results of the individual classification networks

	Raw images			Auto-segmented images				Manually segmented images			
	AUC (95% CI)	Sensitivity (95% CI)	Specificity (95% CI)	AUC (95% CI)	P value^*^	Sensitivity (95% CI)	Specificity (95% CI)	AUC (95% CI)	P value^*^	Sensitivity (95% CI)	Specificity (95% CI)
View classification	1	1	1	–		–	–	–		–	–
diseases detection	0.98 (0.94–0.99)	94.0% (85.3–98.7%)	91.6% (84.6–96.1%)	0.97 (0.93–0.99)	0.57	90.0% (78.2–96.7%)	93.5% (87.0–97.3%)	0.98 (0.94–0.99)	0.73	94.0% (83.5–98.7%)	91.6% (84.6–96.1%)
Etiology identification (average)	0.91	89.3%	79.2%	0.89	–	86.5%	80.0%	0.95	–	89%	88.0%
HCM^a^	0.90 (0.82–0.95)	84.1% (70.0–93.4%)	85.7% (74.6–93.3%)	0.90 (0.82–0.95)	0.99	81.8% (67.3–91.8%)	81.0% (69.1–89.9%)	0.95 (0.89–0.98)	0.06	91.0% (78.3–97.5%)	88.9% (78.4–95.4%)
CA^b^	0.94 (0.88–0.98)	100% (86.8–100%)	74.7% (63.1–83.2%)	0.93 (0.87–0.97)	0.79	88.5% (69.8–97.6%)	92.6% (84.6–97.2%)	0.96 (0.90–0.99)	0.44	92.3% (74.9–99.1%)	86.6% (77.0–93.0%)
HHD^c^	0.88 (0.80–0.93)	83.8% (68–93.8%)	77.1% (65.6–86.3%)	0.85 (0.77–0.91)	0.60	89.2% (74.6–97.0%)	65.7% (54.3–76.7%)	0.93 (0.86–0.97)	0.10	83.8% (68.0–93.8%)	88.6% (78.7–94.9%)

^a^Hypertrophic cardiomyopathy, ^b^cardiac amyloidosis, ^c^hypertensive heart disease, ^*^Compared with the AUC of Raw images

**Fig. 4:**
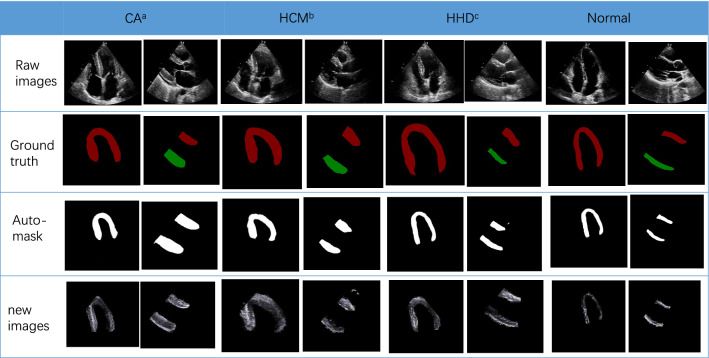
The masks created by manual annotation and auto-segmentation, and the newly generated images of left ventricular myocardium. ^a^cardiac amyloidosis, ^b^hypertrophic cardiomyopathy, ^c^hypertensive heart disease

**Fig. 5:**
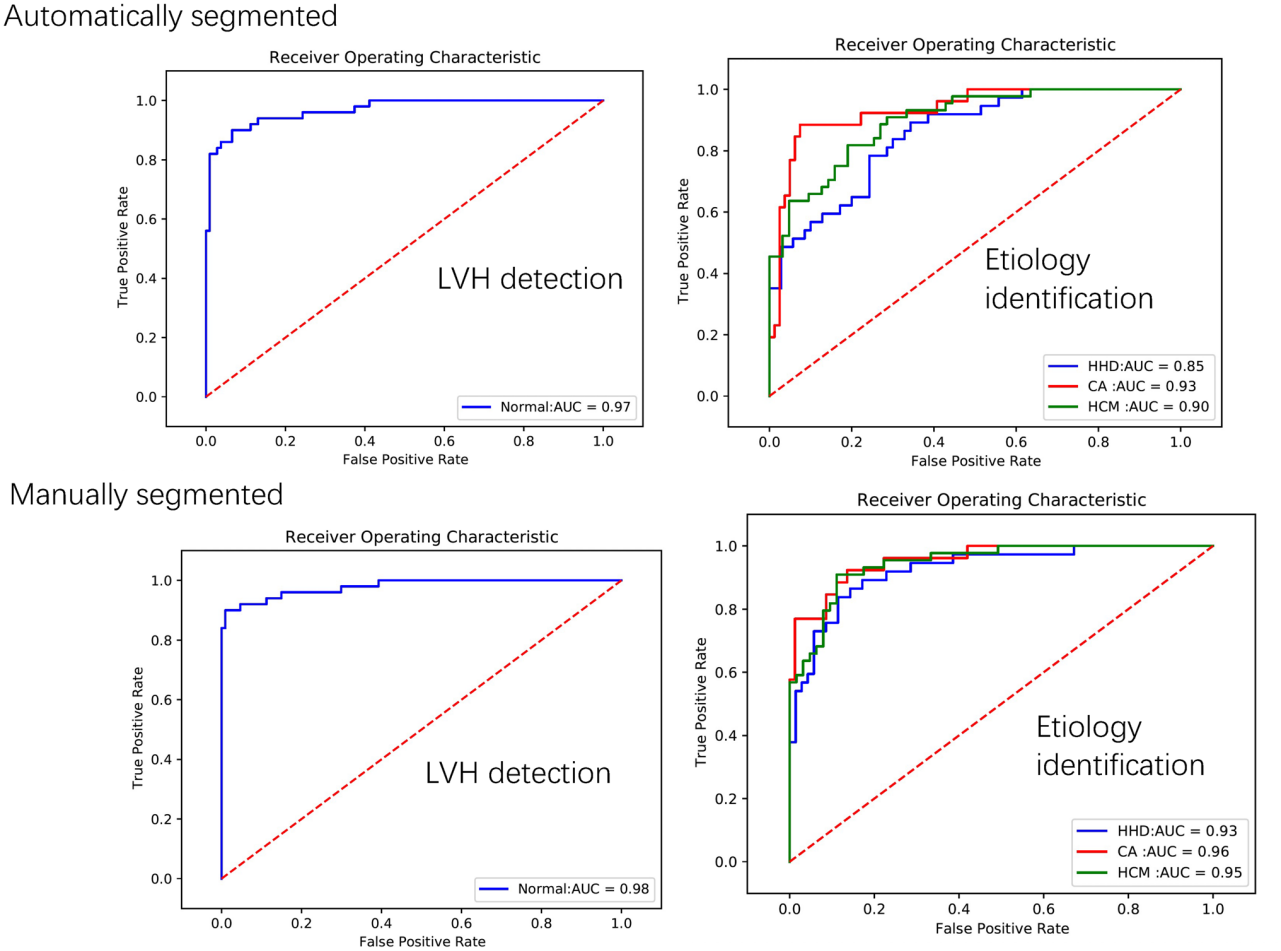
The results of classification networks on automatically or manually segmented images. *LVH* left ventricular hypertrophy, *HCM* hypertrophic cardiomyopathy, *CA* cardiac amyloidosis, *HHD* hypertensive heart disease

The Dice score of the segmentation network was 0.86 ± 0.02, and the IOU, as well as 95%HD were 0.77 ± 0.03, 7.44 ± 7.89 respectively. The newly produced images by segmentation network were used to train the LVH detection and etiology identification models (Fig. 4). In terms of the performance of the LVH detection model trained on auto-segmented images, the AUC was 0.97 (95% CI 0.93–0.99) (Fig. 5) which was comparable to the results of the original images(0.98 vs 0.97, P = 0.57). As for the test results for differentiating the three diseases, the AUC produced by the etiology identification network trained on auto-segmented images were not significantly different from the results on the original images (HCM: 0.90 vs 0.90, P = 0.99; CA: 0.93 vs 0.94, P = 0.79; HHD: 0.85 vs 0.88, P = 0.60) (Fig. 5; Table 2). The accuracy for the LVH detection network and etiology identification network were 92.3% and 74.7%, respectively.

Considering that our segmentation network did not achieve great results with a Dice score of 0.86 and IOU of 0.77, the classification results of the segmented images may be influenced by the performance of the segmentation model. Therefore, we also used manually segmented images (with accurate segmentation) to train the two classification models. The accuracy of LVH identification network was 96.2%. The AUC of recognizing LVH based on manually segmented images was 0.98 (95% CI 0.94–0.99). As for the results of etiology identification, the AUCs produced by this network improved, although not significantly, when compared to the results generated by the network trained on raw images (HCM: 0.95 vs 0.90, P = 0.06; CA: 0.96 vs 0.93, P = 0.44; HHD: 0.93 vs 0.88, P = 0.10), and the accuracy increased from 75.7 to 80.4%. (Fig. 5; Table 2). The remaining results of classification with segmentation were shown in Table 2.

**Fig. 6:**
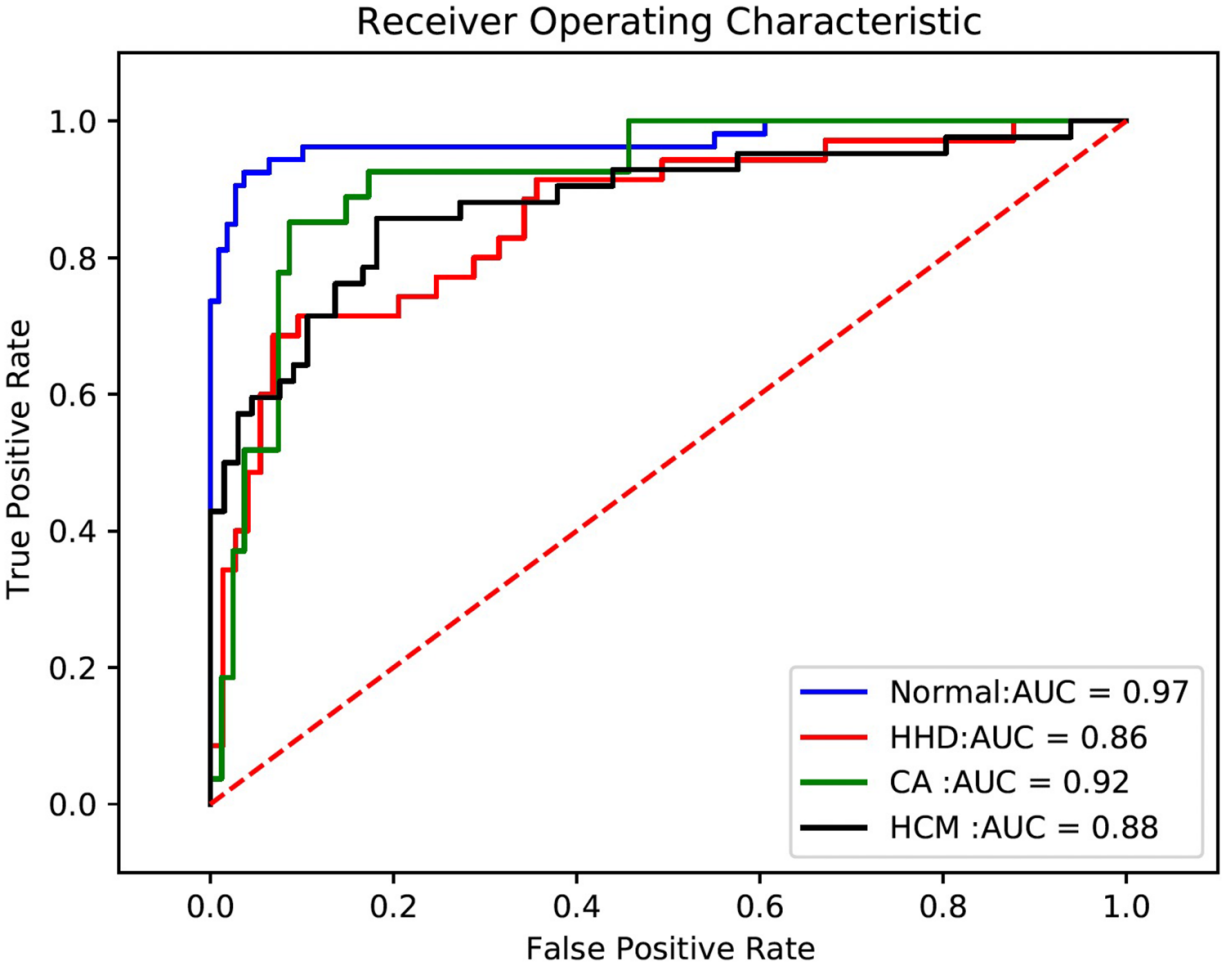
The results of the final integrated framework. *HCM* hypertrophic cardiomyopathy, *CA* cardiac amyloidosis, *HHD* hypertensive heart disease

Given that the performance of the classification models trained on automatically segmented images did not improve when compared with the original images, we did not include the segmentation network in our final architecture. Ultimately, we connected the view classification model, LVH detection model, and etiology identification model to form our integrated framework. The input of this network were pairs of images, and the final output was one of four labels: Normal, HCM, HHD, and CA. The average AUC of this joint network was 0.91 (Fig.6; Table 3), and the average sensitivity and specificity were 83.7% and 90.0% (Table 3), respectively.

**Table 3:** Results of final framework on original images

	AUC (95% CI)	Sensitivity (95% CI)	Specificity (95% CI)
Average	0.91	83.7%	90.0%
Normal	0.97 (0.93–0.99)	92.5% (81.8–97.9%)	96.3% (90.9–99.0%)
HCM^a^	0.88 (0.80–0.93)	85.7% (71.5–94.6%)	81.8% (70.4–90.2%)
CA^b^	0.92 (0.85–0.96)	85.2% (66.3–95.8%)	91.4% (83.0–96.5%)
HHD^c^	0.86 (0.78–0.92)	71.4% (53.7–85.4%)	90.4% (81.2–96.1%)

^a^Hypertrophic cardiomyopathy, ^b^cardiac amyloidosis, ^c^hypertensive heart disease

The accuracy of differentiating normal cardiac structure from LVH by two echocardiographers was 84.6%, and 55.2% for recognizing the etiology of LVH.

**Fig. 7:**
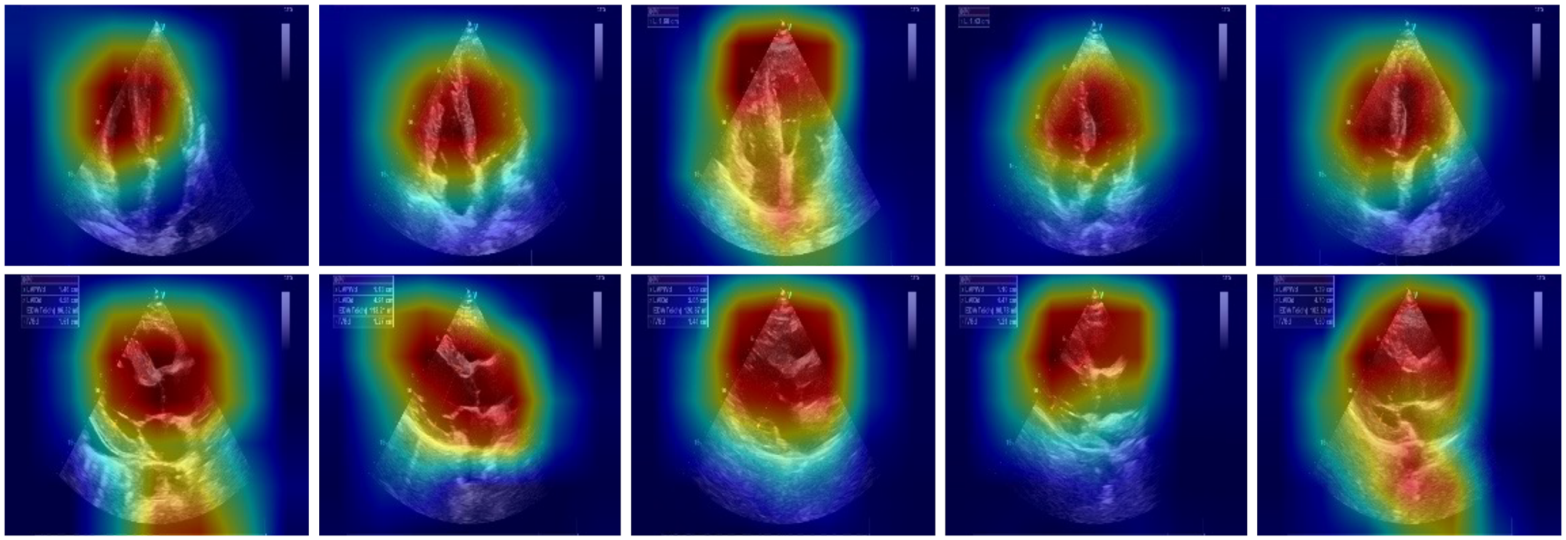
Class activation maps. The heatmaps created by Grad-CAM method highlight the regions which mainly influence the decisions made by the network

As for the interpretability of this network, we utilized the Grad-CAM approach to create heat maps, as shown in Fig. 7, highlighting the region of interest (red) on which the network focused to predict the labels. The red region was concentrated on heart for 87.4% and 71.2% of the A4C and PLX views, respectively. Among these percentages, the red region specifically highlighted the ventricle on 77.3% of the A4C images, and the red region was roughly around the interventricular septum on 52.7% of the PLX images.

## Discussion

LVH is a relatively common morphological alteration of the heart in clinical practice and can be detected by multiple imaging modalities, such as electrocardiography echocardiography, and cardiac magnetic resonance imaging. Among them, echocardiography is a widely used approach to detect and differentiate the etiology of LVH. However, echocardiography has some drawbacks, such as low signal–noise ratio, spotty image quality and lower reproducibility. Deep learning is a promising method to address such problems, which has been applied for many types of research pertaining to echocardiography.

Our research mainly focused on classification tasks, and the proposed deep learning framework achieved good results, with average AUC over 0.90 and corresponding sensitivity and specificity over 80%. Regarding relative research work, several studies have utilized deep learning to identify LVH on echocardiograms; however, few studies have explored the etiology of LVH [21, 22]. One study developed multiple independent convolutional neural networks, one of the representative deep learning algorithms, to differentiate HCM and CA from normal structures, respectively. And their result was similar to ours, with the AUC around 0.9. However, they did not further distinguish between HCM and CA, or other types of LVH [15]. Our framework connected multiple independent models to complete different tasks to not only identify the LVH from normal structures but also to distinguish the etiology of LVH.

In comparison to the two echocardiographers, the classification networks outperformed them, especially on identifying the etiology of LVH (accuracy: 75.7% vs 55.2% for the network vs echocardiographers). Though we did not compare with more experienced observers, the encouraging result still showed that the deep learning network could be a promising measure to help echocardiographers improve diagnostic accuracy, especially for those who are less experience.

Segmentation plays a vital role in echocardiographic assessment, for example, predicting ejection fraction and detecting regional wall motion abnormalities [23, 24]. In this study, we introduced the segmentation network to delineate and separate the LV myocardium, aiming to find out the performance of the network on segmented images which contains important information to identify LVH and with relatively salient edge features provided by masks. We expected the segmentation network would help to improve the performance of the classification network. However, the AUCs of the classification networks (LVH detection and etiology classification) trained on automatically segmented images were not significantly different from those using the original images. Likewise, the accuracy was similar between the two networks, 92.4% vs 92.3% (raw images vs auto-segmented images) for LVH detection and 75.7% vs 74.7% (raw images vs auto-segmented images) for etiology identification. We believe that our segmentation network’s less than satisfactory performance influenced the classification results since inaccurate segmentation may cause some LVM information loss. Thus, we used manually segmented images (with accurate segmentation) to train the classification networks. The AUC of LVH identification produced by this network was similar to that of the original images (both were 0.98). However, the AUCs of distinguishing the three diseases improved, and the accuracy of the two classification networks trained on manually segmented images both increased when compared to the results of the original images (LVH detection: 92.4% vs 96.2%, etiology identification: 75.7% vs 80.4%). The results suggested that more accurate segmentation could help improve classification performance. In this study, our segmentation network did not perform well, so we did not include this module in our final network. In our future work, we will try to improve the capability of the segmentation network and connected it to the final network to improve classification performance.

“Black-box” is an inherent aspect of the deep learning algorithm. Unlike traditional machine learning, which mainly depends on manually extracted features (that help to explain the decision-making process), deep learning methods make predictions based on automatically extracted features. Thus, which features the algorithm uses to make decisions is unknown. The unseen nature of the decision-making process of deep learning is called “Black-bx”.

Only a few studies in medicine using deep learning method focused on the interpretability of the “Black-box” [25, 26]. In our study, we tried to figure out which part of the echocardiography did the network focused on when the model made the decisions. We applied the Grad-CAM method to create class activation maps which highlighted the interested regions, and the network mainly predicted the labels based on the features extracted from that area. As shown in the Fig. 7, whether on A4C or PLX, the highlighted area (red) was major located on heart, not other irrelevant regions, which means the network mainly made the decisions based on the features extracted from heart. In addition, the red area was concentrated on the ventricle in most of the echocardiography on A4C view, and on PLX view, although the ventricle was not specifically highlighted, the red area was roughly around interventricular septum, which further increased the reliability and interpretability of the proposed deep learning network. The “black-box” is a key issue of deep learning algorithms, which might limit the application of this state-of-the-art approach in clinical work [27]. More research adopting deep learning algorithms should focus on both the performance and interpretability of the network.

The architecture we proposed was built on images only. Combining other modalities or clinical measurements may further improve the performance of the network [28], especially 12-lead electrocardiography (ECG), which is another easily available and widely used method for detecting LVH. Numerous studies have shown that ECG-based artificial intelligence methods have the ability to detect LVH [29, 30]. In addition, ECG also contains some information that can help to distinguish the etiology of LVH, for example, if the voltage on ECG is lower than normal under conditions of ventricular wall thickness, then CA should be highly suspected [18]. In future research efforts in the field of cardiovascular imaging, more comprehensive deep learning frameworks involving rapid acquisition, less operator-dependent, and multifaceted information should be considered.

### Limitations

Our study has several limitations. First, the framework was trained with relatively small datasets. Although we included over 700 patients, only two images of each patient were collected, resulting in less than 2000 images. Therefore, further studies should be done with larger sample size, not only with more echocardiograms but also covering a larger number of patients. Second, all the echocardiograms were collected from one medical center, though we collected data from two commonly used ultrasound vendors, the lack of external data influenced the generalizability of this network. So multi-center and multi-vendor studies are needed to improve and test the generalizability of the deep learning model. Third, half of the CA patients have multiple pairs of images (from different inspection times), although we avoided distributing these images from the same patient into different datasets, it may still influence the result of classification results of CA. Fourth, this study did not include other types of LVH, except for HCM, CA, and HHD.

## Conclusion

Deep learning architectures have the ability to automatically detect LVH and even distinguish the underlying etiology of LVH. With sufficient data and appropriate network architecture, this cutting-edge technology has great potential to be applied in clinical practice to assist echocardiographers in making faster and more accurate diagnoses of LVH, especially for less experienced sonographers. More research is needed in the cardiovascular field to help advance the future application of this state-of-the-art technology.

### Supplementary Information

Below is the link to the electronic supplementary material.

Supplementary file1 (PDF 732 kb). Online Fig.1 The loss and accuracy curves of training and validation on original images. Online Fig.2 The loss and accuracy curves of training and validation on auto-segmented images. Online Fig.3 The loss and accuracy curves of training and validation on manually segmented images

## Data Availability

The code of this study is available at https://data.mendeley.com/datasets/537jgxk7hv/1.
