# Supplementary material for: Using deep learning method to identify left ventricular hypertrophy on echocardiography
Source: Int J Cardiovasc Imaging. 2021 Nov 10;38(4):759–69. doi: 10.1007/s10554-021-02461-3 (PMC11130004; doi:10.1007/s10554-021-02461-3)

Table. S1 Baseline characteristics of patients between training, validation and test datasets of control group				
	**training**	**validation**	**test**	**P value**
**No. of Patient**	156	53	50	-
**No. of cases**	156	53	50	-
**Male**	74.5%	67.9%	64%%	0.30
**Age (years)**	57.9±14.4	61.5±11.9	57.8±11.8	0.22
**BSA[a](m^2)**	1.69±0.16	1.66±0.12	1.66±0.12	0.28
**IVSd[b] (cm)**	0.95±0.11	0.96±0.11	0.97±0.12	0.49
**LVPWd[c] (cm)**	0.92±0.12	0.94±0.11	0.93±0.12	0.58
**IVSd/LVPWd**	1.05±0.12	1.03±0.11	1.04±0.11	0.13
**LVM[d](g)**	147.6±32.3	148.7±29.5	146.4±31.6	0.94
**LVMI[e]**	87.1±14.8	89.4±14.4	88.0±15.3	0.63
**EF[f] (%)**	65.8±6.6	64.1±7.9	63.8±6.7	0.13
**LVDd[g] (cm)**	4.60±0.39	4.64±0.39	4.57±0.43	0.63

[a]BSA: Body surface area; [b]IVSd: Diastolic interventricular septum; [c]LVPWd: Diastolic left ventricular post wall; [d]LVM: Left ventricular mass; [e]LVMI: Left ventricular mass index; [f]EF: Ejection fraction; [g]LVDd: Diastolic left ventricular diameter;

Table. S2 Baseline characteristics of patients between training, validation and test datasets of hypertrophic cardiomyopathy group				
	**training**	**validation**	**test**	**P value**
**No. of Patient**	128	46	44	-
**No. of cases**	129	47	44	-
**Male**	64.8%	67.4%	68.2%	0.90
**Age (years)**	55.6±14.5	57.8±14.6	58.6±11.6	0.38
**BSA[a](m^2)**	1.56±0.15	1.72±0.13	1.70±0.12	0.44
**IVSd[b] (cm)**	1.98±0.56	2.06±0.53	1.89±0.48	0.30
**LVPWd[c] (cm)**	1.11±0.24	1.09±0.18	1.11±0.22	0.84
**IVSd/LVPWd**	1.85±0.61	1.94±0.52	1.77±0.61	0.39
**LVM[d](g)**	280.9±92.4	296.0±83.9	277.3±66.7	0.51
**LVMI[e]**	166.3±52.5	172.6±50.3	164.1±40.9	0.68
**EF[f] (%)**	69.8±8.4	69.2±5.3	68.9±7.6	0.79
**LVDd[g] (cm)**	4.37±0.55	4.43±0.50	4.48±0.45	0.45
**LVOT[h] (mmHg)**	31.2±43.6	31.9±45.6	36.0±40.3	0.86

[a]BSA: Body surface area; [b]IVSd: Diastolic interventricular septum; [c]LVPWd: Diastolic left ventricular post wall; [d]LVM: Left ventricular mass; [e]LVMI: Left ventricular mass index; [f]EF: Ejection fraction; [g]LVDd: Diastolic left ventricular diameter; [h]LVOT: Left ventricular outflow tract;

Table. S3 Baseline characteristics of patients between training, validation and test datasets of HHD group				
	**training**	**validation**	**test**	**P value**
**No. of Patient**	114	38	37	-
**No. of cases**	114	38	37	-
**Male**	86.8%	71.1%	78.4%	0.16
**Age (years)**	63.8±11.3	55.6±14.3	55.8±13.3	<0.05
**BSA[a](m^2)**	1.70±0.16	1.71±0.15	1.67±0.12	0.38
**IVSd[b] (cm)**	1.30±0.13	1.37±0.17	1.34±0.14	0.01
**LVPWd[c] (cm)**	1.13±0.14	1.20±0.17	1.19±0.19	0.02
**IVSd/LVPWd**	1.16±0.10	1.15±0.10	1.14±0.10	0.69
**LVM[d](g)**	219.6±86.9	239.0±91.6	221.1±75.6	0.47
**LVMI[e]**	132.8±42.8	143.2±50.3	136.8±43.8	0.46
**EF[f] (%)**	67.2±8.7	63.5±9.7	66.5±7.2	0.083
**LVDd[g] (cm)**	4.77±0.61	4.77±0.64	4.61±0.45	0.35

[a]BSA: Body surface area; [b]IVSd: Diastolic interventricular septum; [c]LVPWd: Diastolic left ventricular post wall; [d]LVM: Left ventricular mass; [e]LVMI: Left ventricular mass index; [f]EF: Ejection fraction; [g]LVDd: Diastolic left ventricular diameter;

Table. S4 Baseline characteristics of patients between training, validation and test datasets of cardiac amyloidosis group				
	**training**	**validation**	**test**	**P value**
**No. of Patient**	34	13	11	-
**No. of cases**	83	28	26	-
**Male**	70.5%	92.3%	81.8%	0.28
**Age (years)**	62.0±8.0	61.5±10.1	62.6±8.0	0.94
**BSA[a](m^2)**	1.64±0.14	1.78±0.14	1.68±0.14	<0.05
**IVSd[b] (cm)**	1.49±0.21	1.65±0.24	1.5±0.18	0.02
**LVPWd[c] (cm)**	1.35±0.25	1.50±0.32	1.37±0.27	0.04
**IVSd/LVPWd**	1.11±0.12	1.12±0.11	1.12±0.17	0.98
**LVM[d](g)**	227.6±52.9	276.1±82.6	231.3±47.0	<0.05
**LVMI[e]**	139.3±32.2	153.8±40.1	138.7±32.7	0.13
**EF[f] (%)**	61.2±7.1	48.8±12.3	57.3±12.6	<0.05
**LVDd[g] (cm)**	4.15±0.42	4.22±0.43	4.16±0.48	0.76
**GLS-Avg[h] (%)**	14.0±3.9	10.7±3.6	11.4±4.2	<0.05

[a]BSA: Body surface area; [b]IVSd: Diastolic interventricular septum; [c]LVPWd: Diastolic left ventricular post wall; [d]LVM: Left ventricular mass; [e]LVMI: Left ventricular mass index; [f]EF: Ejection fraction; [g]LVDd: Diastolic left ventricular diameter; [h]GLS-Avg: Average global longitudinal strain;

online Figure 1

Etiology classification model

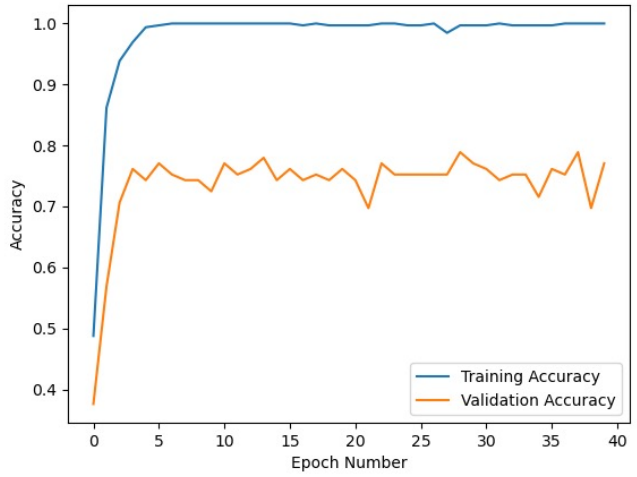

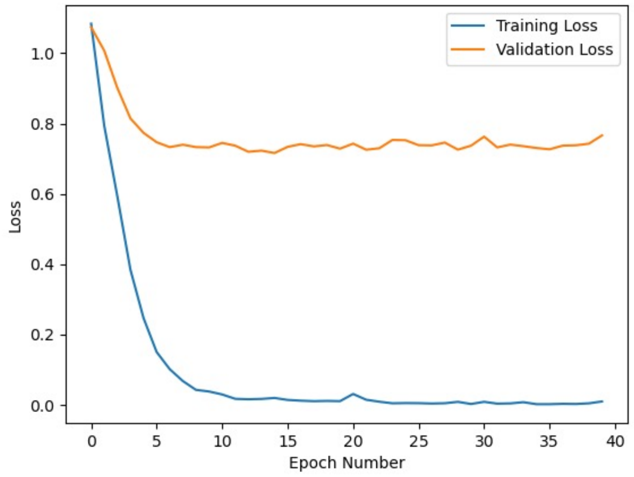

LVH detection model

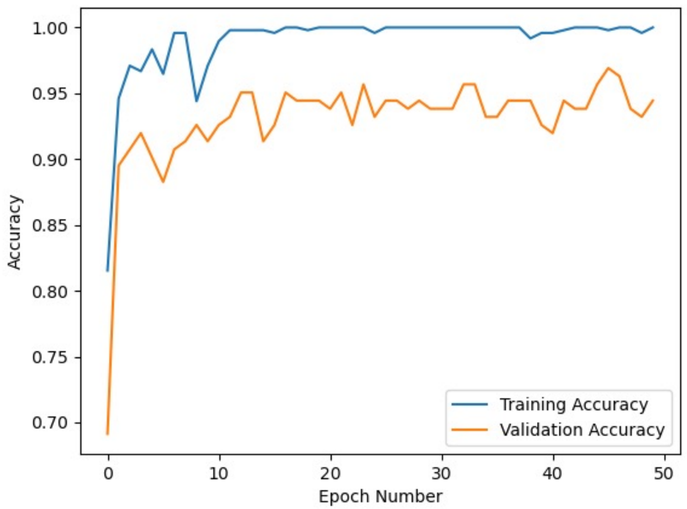

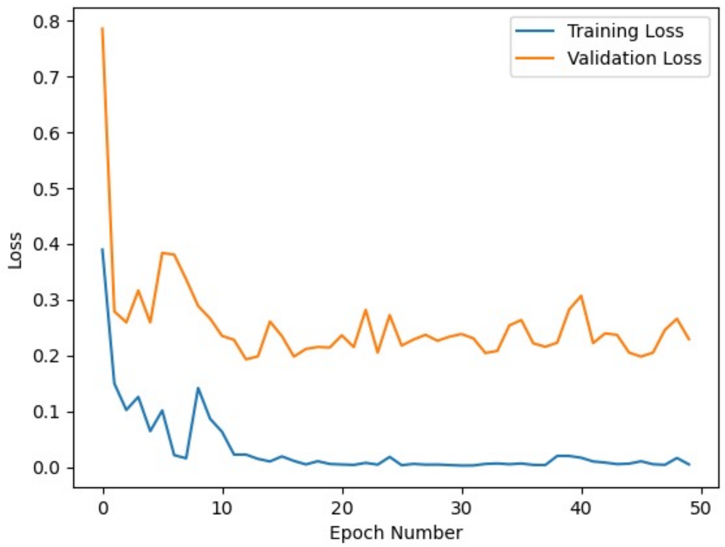

online Figure 2

Etiology identification model

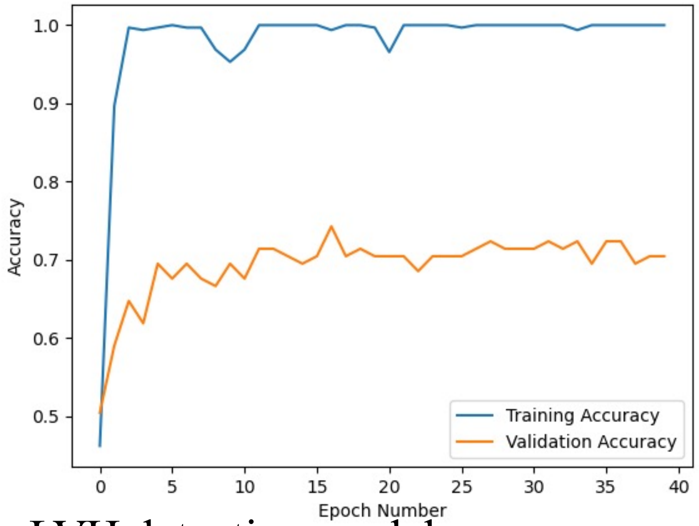

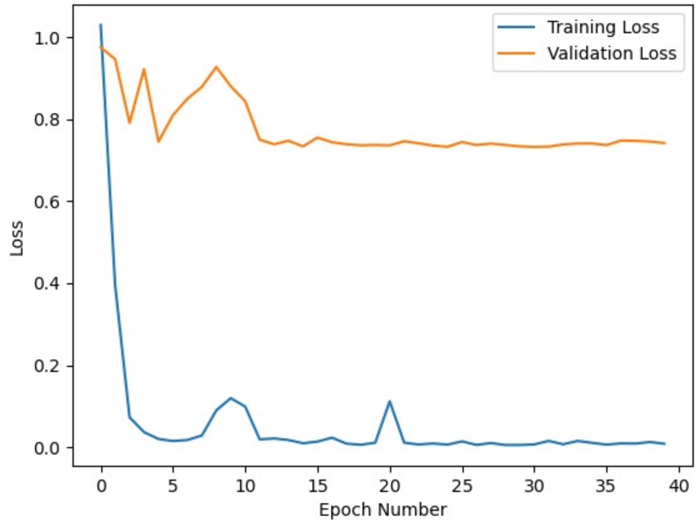

LVH detection model

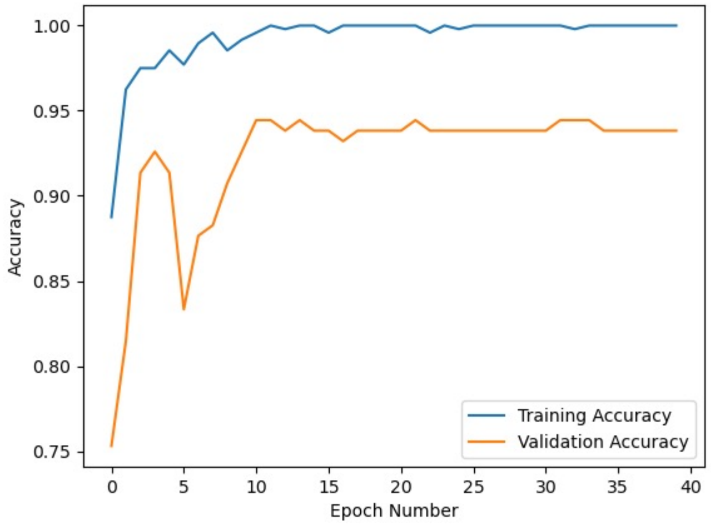

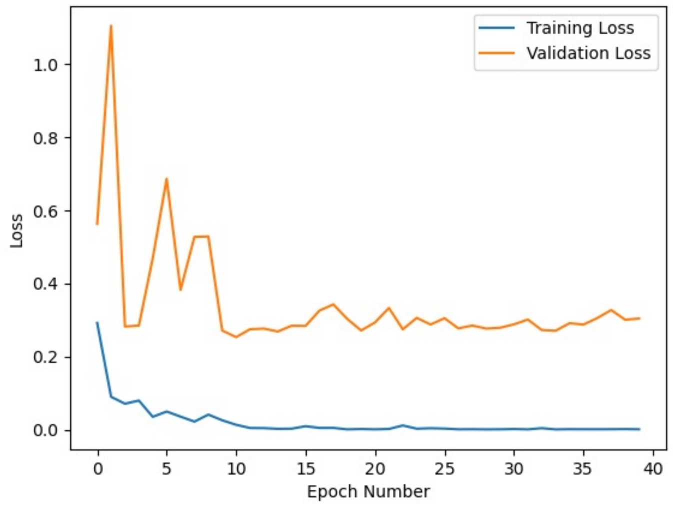

online Figure 3

Etiology classification model

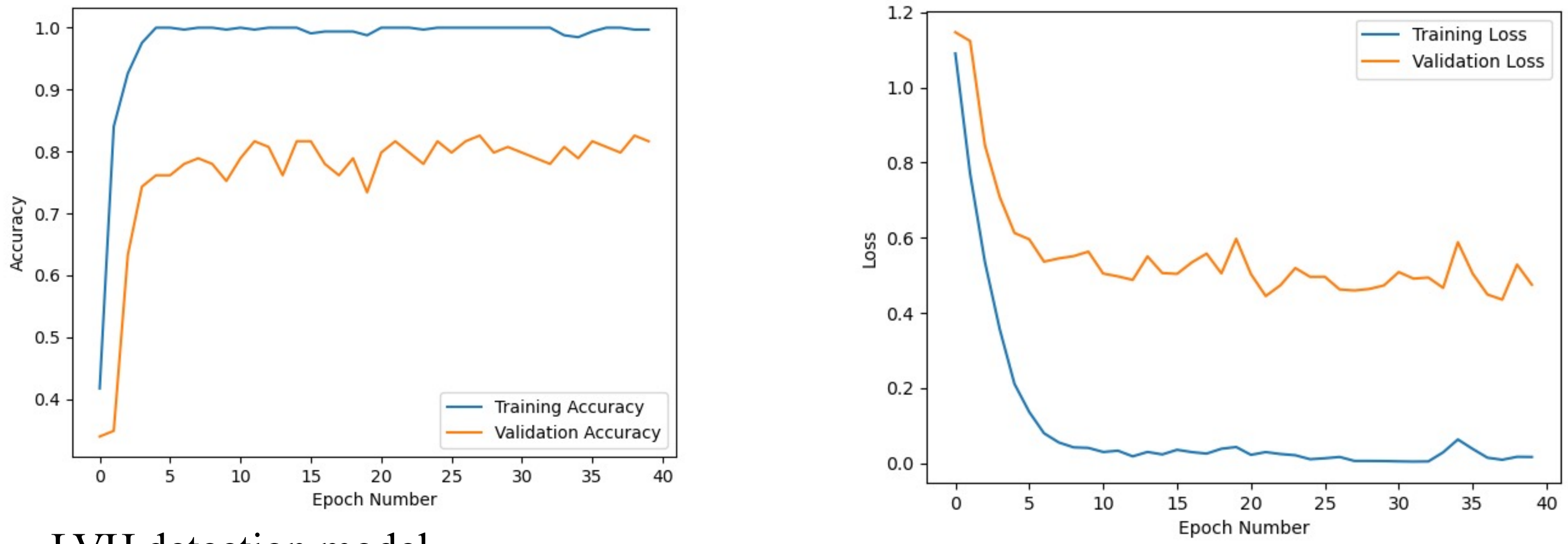

LVH detection model

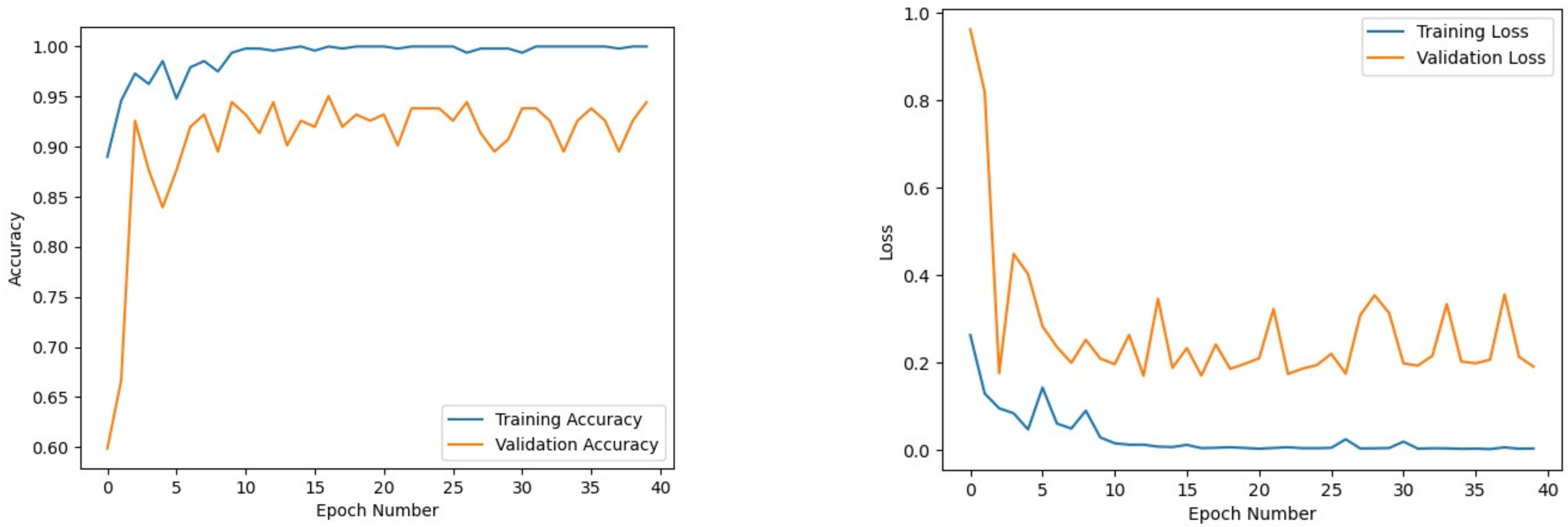

Supplement: Supplementary file 1 — Supplementary file1 (PDF 732 kb). Online Fig.1 The loss and accuracy curves of training and validation on original images. Online Fig.2 The loss and accuracy curves of training and validation on auto-segmented images. Online Fig.3 The loss and accuracy curves of training and validation on manually segmented images [file 10554_2021_2461_MOESM1_ESM.pdf]
